# Physiology of the volume-sensitive/regulatory anion channel VSOR/VRAC: part 2: its activation mechanisms and essential roles in organic signal release

**DOI:** 10.1186/s12576-024-00926-3

**Published:** 2024-06-14

**Authors:** Yasunobu Okada

**Affiliations:** 1 National Institute for Physiological Sciences (NIPS), 5-1 Higashiyama, Myodaiji, Okazaki, Aichi 444-8787 Japan; 2 Department of Integrative Physiology, Graduate School of Medicine, Akita University, Akita, Japan; 3 Department of Physiology, School of Medicine, Aichi Medical University, Nagakute, Japan; 4 Graduate University for Advanced Studies (SOKENDAI), Hayama, Kanagawa Japan

**Keywords:** Volume-sensitive anion channel, LRRC8, Pore size, Organic signal, ROS, ATP

## Abstract

The volume-sensitive outwardly rectifying or volume-regulated anion channel, VSOR/VRAC, which was discovered in 1988, is expressed in most vertebrate cell types, and is essentially involved in cell volume regulation after swelling and in the induction of cell death. This series of review articles describes what is already known and what remains to be uncovered about the functional and molecular properties as well as the physiological and pathophysiological roles of VSOR/VRAC. This Part 2 review article describes, from the physiological and pathophysiological standpoints, first the pivotal roles of VSOR/VRAC in the release of autocrine/paracrine organic signal molecules, such as glutamate, ATP, glutathione, cGAMP, and itaconate, as well as second the swelling-independent and -dependent activation mechanisms of VSOR/VRAC. Since the pore size of VSOR/VRAC has now well been evaluated by electrophysiological and 3D-structural methods, the signal-releasing activity of VSOR/VRAC is here discussed by comparing the molecular sizes of these organic signals to the channel pore size. Swelling-independent activation mechanisms include a physicochemical one caused by the reduction of intracellular ionic strength and a biochemical one caused by oxidation due to stimulation by receptor agonists or apoptosis inducers. Because some organic substances released via VSOR/VRAC upon cell swelling can trigger or augment VSOR/VRAC activation in an autocrine fashion, swelling-dependent activation mechanisms are to be divided into two phases: the first phase induced by cell swelling per se and the second phase caused by receptor stimulation by released organic signals.

**Supplementary Information:**

The online version contains supplementary material available at 10.1186/s12576-024-00926-3.

## Introduction

Mammalian anion channels are known to be classified into six major groups: ligand-gated receptor-coupled, voltage-gated ClC-type, cyclic AMP/PKA-activated cystic fibrosis transmembrane conductance regulator (CFTR), Ca^2+^-activated TMEM16/ANO, acid-activated ASOR/PAC, and swelling-activated anion channels (see Review [1]). The last one, called the volume-activated anion channel (VAAC), is involved in cell volume regulation and consists of two types, the intermediate-conductance outwardly rectifying anion channel, which was termed the volume-sensitive outwardly rectifying anion channel (VSOR) or volume regulated anion channel (VRAC) (see Review [2]), and the large-conductance ohmic maxi-anion channel (Maxi-Cl) (see Review [3]).

The intermediate conductance one, here called the volume-sensitive outwardly rectifying/volume-regulatory anion channel (VSOR/VRAC), was first functionally found in 1988 independently by two groups ([4, 5]). Soon thereafter, its phenotypical properties were well-characterized [6–8], but the molecular nature remained unknown for over a quarter of a century. In recent years, the pore-forming core component and the swelling-sensing subcomponents of VSOR/VRAC were identified as LRRC8 members in 2014 [9, 10] and TRPM7 in 2021 [11], respectively, as summarized in the Part 1 article [12].

VSOR/VRAC is known to be activated not only by cell swelling but also by some other physicochemical and biochemical stimuli even in the absence of cell swelling. In addition, accumulating evidence showed that VSOR/VRAC plays not only the volume-regulatory role but also other roles including mediation of organic signal release, induction of apoptotic, necrotic, and pyroptotic cell death, and acquirement of anti-cancer drug resistance. Thus, here in this Part 2 article, from physiological and pathophysiological standpoints, I review how VSOR/VRAC is involved in the cellular release of autocrine/paracrine organic signals, and how it is activated, in a swelling-dependent and -independent manner, together with pointing out what remains to be elucidated in future studies. The VSOR/VRAC roles in cell death induction and acquisition of anti-cancer drug resistance will be reviewed in the next Part 3 article.

## Mediation of release of organic substances and signals via VSOR/VRAC

Since organic solutes produced within cells are intracellularly accumulated, and their extracellular concentrations are negligibly low under ordinal conditions, they are readily driven out of cells by such chemical potential (concentration) gradients when some diffusional channel-mediated routes are available. For the release of negatively charged organic solutes, the intracellular negative potential is to be added to the driving force. For example for ATP^4−^, the electrochemical potential gradient reaches the order of 10^10^ when the intracellular electrical potential is around − 60 mV [13].

After the establishment of VSOR/VRAC role in cell volume regulation, its mediation of the cellular release of small organic substances was found to be another important role of VSOR/VRAC in the early 1990s, as summarized by Strange et al. [6, 14]. Thereafter, this VSOR/VRAC role was supported by the functional and structural evaluation of the pore size of VSOR/VRAC. Recent studies have elucidated the physiological/pathophysiological significance of VSOR/VRAC-mediated release of organic substances as paracrine/autocrine signals.

### Swelling-activated release of intracellular organic solutes and the pore size of VSOR/VRAC

Swelling-activated Na^+^-independent (that is, Na^+^-driven cotransport-independent) release of intracellular glutamate, aspartate, and taurine from mammalian cells was, for the first time, observed in rat astrocytes in 1990 by Kimelberg et al. [15]. In 1991, hypotonicity-induced, Na^+^-independent taurine release was found to be sensitive to a Cl^−^ channel blocker DIDS in rabbit lymphocytes [16]. Then, similar Na^+^-independent release of glutamate, taurine, and glycine from canine kidney MDCK cells was shown to be linearly dependent on these concentrations in 1992 [17], suggesting the involvement of diffusional channel-mediated, but not saturable carrier/transporter-mediated, transport which is activated by cell swelling. The swelling-activated, VSOR/VRAC-mediated currents conveyed by negatively charged organic substances were, in fact, recorded under voltage-clamp and bi-ionic conditions for gluconate in human epithelial Intestine 407 cells under the whole-cell configuration by Kubo and Okada [18] and for aspartate, glutamate, and taurine, which is a zwitter ion and electrically neutral at physiological pH but becomes negatively charged at alkaline pH, in MDCK cells in single-channel recording modes by Banderali and Roy [19] also in 1992. These patch-clamp studies evaluated their permeability coefficient: P_aspartate_/P_Cl_ ~ 0.2 [17], P_gluconate_/P_Cl_ ~ 0.1 [18], P_glutamate_/P_Cl_ ~ 0.2 [19], and P_taurine_/P_Cl_ ~ 0.75 [19]. Because VSOR/VRAC is a low-field anion channel, as described in the Part 1 article [12], it is reasonable that the sequence of these permeability coefficients (chloride > taurine > aspartate ~ glutamate > gluconate) is in fairly good accordance with the sequence for their effective diameters listed in Table 1. Also, it must be noteworthy that all these organic substances have much smaller diameters [13, 14, 20] than a pore diameter of VSOR/VRAC (7–13 Å) functionally estimated by three different and unrelated techniques by Nilius and Droogmans [21], by Droogmans et al. [22], and by Ternovsky et al. [23] (Table 2A).

**Table 1:** Effective diameters of some inorganic and organic anions and osmolytes that potentially permeate VSOR/VRAC channels

Anion/osmolyte	Effective diameter (Å)^a^	References
Cl^−^	3.6	[20]
Glycine (C_2_H_5_NO_2_)	4.2	[177]
NO_3_^−^	4.3	[20]
Taurine (C_2_H_7_NO_3_S)	5.3	[14]
HPO_4_^2−^	5.5	[13]
Proline (C_5_H_9_NO_2_)	5.6	[14]
Betaine (C_5_H_11_NO_2_)	5.7	[14]
*Myo*-inositol (C_6_H_12_O_6_)	6.1	[14]
Aspartate^−^ (C_4_H_7_NO_4_)	6.8	[13]
Glutamate^−^ (C_5_H_9_NO_4_)	6.9	[20]
Gluconate^−^ (C_6_H_12_O_7_)	7.0	[13]
UTP^4−^ (C_9_H_15_N_2_O_15_P_3_)	10.7	[13]
Glutathione^−^ (C_10_H_17_N_3_O_6_S)	10.8	[92]
ADP^3−^ (C_10_H_15_N_5_O_10_P_2_)	10.9	[13]
ATP^4−^ (C_10_H_16_N_5_O_13_P_3_)	11.5	[178]
Mg-ATP^2−^	12.0	[178]
cGAMP^2−^ (C_20_H_24_N_10_O_13_P_2_)	12.0	[179]

^a^The unhydrated diameter was calculated as a geometric mean of three dimensions according to the formula: (L_1_ × L_2_ × L_3_)^1/3^ where L_1_, L_2_, and L_3_ are the length, width, and thickness, respectively, of the molecule

**Table 2:** Comparison between the VSOR/VRAC pore sizes estimated by electrophysiological and cryo-microscopical methods

A. Evaluation method	Functional pore diameter (Å)	References
Cut-off size of permeant organic anions	11	[21]
Cut-off size of basket-shaped permeant blockers	7.3–11.5	[22]
Non-electrolyte partitioning	12.6	[23]

B. LRRC8 multimer	Structural pore diameter (Å)^a^	References
LRRC8A homohexamer	2	[30]
″	4	[31]
″	5.8	[28]
″	6.6	[32]
″	7.6	[29]
LRRC8C homoheptamer	12	[33]
LRRC8C-8A(IL1^25^) homoheptamer^b^	9.4	[35]
LRRC8D homohexamer	11.5	[27]
LRRC8A/8C heterohexamer	6.0	[33]
″	4.2	[34]

A. Functional diameters of the pore estimated by three different approaches

B. Structural diameters of the narrowest constriction portion of the pore of multimeric LRRC8 channels determined by cryo-EM microscopy

^a^The values may have fluctuated depending on the experimental conditions especially employed ionic strength and lipid environments

^b^LRRC8C-8A(IL1^25^) represents a chimera comprising LRRC8C and 25 amino acids unique to the first intracellular loop (IL1) of LRRC8A

Taking the capability of VSOR/VRAC to serve as the pathway for the swelling-induced release of intracellular organic substances into consideration, this volume-sensitive anion channel has sometimes been also called the volume-sensitive organic osmolyte and anion channel (VSOAC) [6, 14]. However, it must be noted that not only VSOR/VRAC but also Maxi-Cl and CFTR can provide the pathways for the release of organic solutes such as glutamate, ATP, and GSH, as pointed out in our previous article [1].

In agreement with the fact that LRRC8A represents an indispensable core molecule of VSOR/VRAC [9, 10], LRRC8A was found to be a prerequisite to the hypotonicity-induced release of organic osmolytes mediated by VSOR/VRAC. Gene knockout of LRRC8A abolished the release of taurine from HCT116 cells [10] and HEK293 cells [24, 25], that of glutamate from HEK293 cells [25] and rat astrocytes [26], and that of aspartate, lysine, serine, GABA, and *myo*-inositol from HEK293 cells [25]. Gene knockout experiments also showed that LRRC8D is essential for swelling-induced taurine release from HEK293 cells [24]. LRRC8A/8D heteromers can transport relatively large organic anions such as glutamate^−^ and aspartate^−^ as well as uncharged organic osmolytes, taurine, *myo*-inositol, and GABA, and even positively charged lysine^+^ [25]. In contrast, LRRC8A/8C and LRRC8A/8E heteromers conduct Cl^−^ and aspartate^−^ but are much less permeable to GABA and *myo*-inositol [25]. Thus, it is concluded that LRRC8D makes VSOR/VRAC more permeable to larger organic anions and uncharged or cationic organic osmolytes. These results are in good agreement with the 3D structures analyzed by cryo-electron microscopy (cryo-EM). The narrowest constriction part (at the selectivity filter) of the pore of LRRC8D homohexamers (at F143) [27] is much larger than that of LRRC8A homohexamers (at R103) [28–32], LRRC8A/8C heterohexamers (at R103/L105) [33, 34], and LRRC8C-8A(IL1^25^) homoheptamers (at L105) [35] but is comparable to that of LRRC8C homoheptamers (at L105) [33], as listed in Table 2B. How large the pores of LRRC8A/8D and LRRC8A/8E heteromers awaits cryo-EM studies in the immediate future.

### VSOR/VRAC-mediated transport of organic signaling molecules

**Fig. 1:**
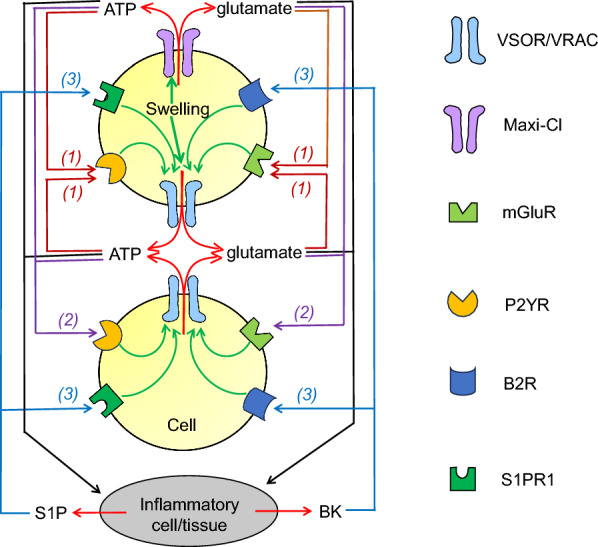
Autocrine/paracrine signaling roles of glutamate and ATP released via VSOR/VRAC and Maxi-Cl upon cell swelling in VSOR/VRAC activation. First (*(1): brown arrows*), such released glutamate and ATP activate, in an autocrine fashion, VSOR/VRAC via stimulation of GPCRs (mGluR and P2YR) in a cell in response to osmotic swelling. Second (*(2): violet arrows*), glutamate and ATP then activate, in a paracrine fashion, VSOR/VRAC via stimulation of GPCRs in another neighboring cell even in the absence of swelling. These glutamate and ATP may also trigger (*black arrows*), in a paracrine fashion, induction of inflammation in the surrounding cells/tissues from which BK and S1P are thereafter released. Third (*(3): blue arrows*), BK and S1P then activate VSOR/VRAC via stimulation of their receptors (B2R and S1PR1). (See text for details.)

In addition to volume-regulatory roles, VSOR/VRAC plays roles in the transmission of paracrine/autocrine signaling by transporting numbers of negatively charged organic substances, such as glutamate^–^, aspartate^–^, ATP^4–^, glutathione (GSH^–^), itaconate^2–^, and 2′3′-cyclic-GMP-AMP (cGAMP^2–^). Moreover, glutamate and ATP released via VSOR/VRAC were shown to activate VSOR/VRAC in a positive feedback fashion through stimulation of their receptors (Fig. 1).

#### VSOR/VRAC-mediated glutamate release

Kimelberg et al. [15] found that the swelling-induced release of glutamate, aspartate, and taurine from rat astrocytes in culture is sensitive to many known anion transport blockers. Also, Roy and Malo [17] observed that large losses of amino acids, such as glutamate, taurine, and glycine, take place during cell volume regulation upon a hypotonic challenge to canine MDCK cells through diffusional routes rather than transporters/carriers. Then, Banderali and Roy [19], for the first time, showed that an outwardly rectifying anion channel provides the diffusional pathway for the swelling-induced release of some intracellular organic substances including glutamate. Since some of these organic substances are known to play signaling roles as excitatory amino acids between neurons and between glial and neuronal cells in the brain under physiological/pathological situations, the roles of VSOR/VRAC in glutamate release mainly from glial cells were thereafter studied.

Electrophysiological and pharmacological evidence was provided for the VSOR/VRAC role in glutamate release from mouse astrocytes induced by hypoosmotic and ischemic stress [36]. Accordingly, DCPIB, which is a relatively most VSOR/VRAC-specific blocker among available anion channel blockers (see Reviews [1, 12]), was shown to inhibit osmotic swelling-induced glutamate release from rat primary astrocytes [37] and human retinal glial Müller MIO-M1 cells [38] as well as the hypotonicity-induced release of aspartate, which is a non-metabolized analog of glutamate, from rat astrocytes [37, 39]. However, DCPIB was unexpectedly found to inhibit not only glutamate release mediated by VSOR/VRAC but also that mediated by connexin hemichannels as well as glutamate uptake via glutamate transporter GLT1 in rat glial cells [40].

Molecular evidence for VSOR/VRAC-mediated glutamate/aspartate release from osmotically swollen astrocytes was recently provided by the effects of reduced expression of LRRC8A. First, siRNA-mediated LRRC8A knockdown brought about 70% and 90% inhibition of the release of glutamate and aspartate from rat astrocytes induced by a moderate hypotonic (70% osmolarity) challenge [41, 42] and by a severe hypotonic (30% osmolarity) challenge [42], respectively. Second, astrocyte-specific *Lrrc8a* knockout caused 93% and 70% inhibition of the release of glutamate and aspartate from swollen mouse astrocytes challenged by an intracellular hypertonic (135% osmolarity) solution [26] and an extracellularly hypotonic (70% osmolarity) solution [43], respectively. Third, aspartate release from primary rat astrocytes challenged by hypotonicity (70% osmolarity) was suppressed to ~ 60% and ~ 25% by double knockdown of *Lrrc8c plus Lrrc8e* and by that of *Lrrc8c plus Lrrc8d*, respectively [44]. However, it must be noted that swelling-induced glutamate release from astrocytes is mediated not only by VSOR/VRAC but also by Maxi-Cl channels [36], the latter of which represents another type of VAACs [1]. That is why gene deletion of LRRC8 members failed to cause the complete abolition of swelling-induced glutamate release from astrocytes. Since the core molecule of Maxi-Cl was recently identified as SLCO2A1 [45], we need hereafter to answer the question as to what degree VSOR/VRAC and Maxi-Cl contribute to glutamate release induced by cell swelling each other in the cell types in question under the given conditions.

Extracellular application of ATP was found to stimulate the release of aspartate [41, 46] and glutamate [46, 47] from astrocytes by activating VSOR/VRAC through stimulation of purinergic P2Y receptors (P2YRs) [47, 48] under isotonic conditions. Extracellular ATP-induced aspartate release was shown to be, as a matter of course, inhibited by siRNA-mediated *Lrrc8a* knockdown in rat astrocytes [41]. Extracellular application of glutamate was also shown to induce VSOR/VRAC activation under isotonic conditions through activation of muscarinic glutamate receptor (mGluR) in mouse astrocytes [49]. Thus, glutamate released via both types of VAACs contributes to the activation of VSOR/VRAC via mGluRs in the cell itself and in a nearby cell in autocrine and paracrine fashions (Fig. 1: *right side (1)* and *(2)*), respectively. Since both glutamate and ATP are known to be released from swollen cells via both types of VAACs, Maxi-Cl and VSOR/VRAC, hereafter we need to answer the question as to what extent swelling-induced glutamate release is caused by cell swelling per se and by glutamate and ATP secondary released in the particular cell types under the given conditions.

Exposure to extracellular bradykinin (BK), which is a proinflammatory nine-amino acid peptide, was shown to trigger VSOR/VRAC activation [50, 51] via bradykinin B2 receptor (B2R) and to stimulate glutamate release [50] without exhibiting cell swelling. BK is generated from kininogens by the action of kallikrein, represents an initial mediator of inflammation [52], and is known to be released from injured and inflammatory sites such as the central nervous system after brain trauma and stroke [53, 54]. Since major excitatory neurotransmitter glutamate exerts as signaling and causal factors for inflammation coupled to some disorders in the central system [55] and in the peripheral system [56, 57], glutamate released via VSOR/VRAC and Maxi-Cl may be involved in the induction of cell/tissue inflammation (Fig. 1: *right side, black arrow*) and therefrom secondarily causes BK release thereby inducing B2R-mediated VSOR/VRAC activation (Fig. 1: *right side (3)*) followed by glutamate release, in a positive feedback manner, from the cells stimulated by BK.

Extracellular application of sphingosine-1-phosphate (S1P) was shown to activate VSOR/VRAC via S1P receptor 1 (S1PR1) under isotonic conditions in murine RAW 264.7 macrophages [58]. A pleiotropic lipid mediator S1P plays a significant role in inflammation [59, 60] and is known to be released from the inflamed cells [61–63]. Therefore, VSOR/VRAC may be activated by S1P released from inflammatory cells/tissues (Fig. 1: *left side (3)*), which were caused by exposure to glutamate released via VSOR/VRAC and Maxi-Cl (Fig. 1: *right side, black arrow*), thereby causing VSOR/VRAC activation in the cells stimulated by S1P and therefrom glutamate release in a positive feedback manner.

#### VSOR/VRAC-mediated ATP release

ATP acts as a major messenger molecule for autocrine and paracrine signaling in the extracellular space [13, 64, 65], whereas it serves as an energy source in the cytosol. ATP is released not only by vesicular exocytosis but also by the transport via several non-vesicular pathways including anion channels [13]. In particular, large-conductance Maxi-Cl anion channels have been shown to serve as a major pathway for swelling- and ischemia-induced release of ATP^4–^ from many cell types such as astrocytes and cardiomyocytes [3]. Swelling-induced ATP release was suggested to be mediated also via VSOR/VRAC largely based on pharmacological evidence in bovine aortic endothelial cells [66], mouse DRG neurons [67], and mouse RAW 264.7 macrophages [58]. In contrast, swelling-induced ATP release was not inhibited by a number of VSOR/VRAC blockers in bovine ocular ciliary epithelial cells [68], human epithelial Intestine 407 cells [69], rat cardiomyocytes [70], and mouse astrocytes [71]. ATP release induced by mechanical stimulation was found to be sensitive to VSOR/VRAC blockers but insensitive to *Lrrc8a* knockdown in rat astrocytes [72]. In contrast, gene knockdown of LRRC8A was shown to suppress ATP release induced by hypoosmotic stimulation in HEK293 cells [73], HeLa cells [74], and mouse microglial BV-2 cells [74] and that induced by application of S1P in mouse microglial BV-2 cells [74, 75] and in human breast cancer MCF7 and MDA-MB-231 cells [76]. Collectively, it appears that VSOR/VRAC can mediate ATP release in many, but not all, cell types. Notably, the functional pore diameter of VSOR/VRAC (Table 2A) is very close to the effective diameter of ATP^4‒^ and MgATP^2‒^ (Table 1). Thus, the ATP conductivity of VSOR/VRAC pores may be prone to be affected by alterations in the surrounding microenvironment at the plasma membrane, especially in the lipid microenvironment. Also, the LRRC8 heteromer composition of VSOR/VRAC may affect its ATP conductivity, because ATP release was found to be provoked by hypotonic stimulation in *Xenopus* oocytes when LRRC8A was co-expressed with LRRC8E or LRRC8C but not with LRRC8B or LRRC8D [77]. In light to these observations, we now need to examine to what degree each type of VAACs contributes to swelling-induced ATP release from the concerned cell types under the given conditions. Also, we hereafter need to answer the question as to what degree swelling-induced ATP release is induced by cell swelling per se and by glutamate and ATP secondary released upon cell swelling from the concerned cell types under the given conditions. Furthermore, from now on, we need to pay attention to what extent swelling-induced ATP release is affected by following two opposite autocrine actions in the particular cell types under the given conditions because extracellular ATP exerts two contradictory effects, an open-channel blocking action [78–80] and receptor-mediated swelling-independent activating action [48].

ATP released via both types of VAACs provokes the activation of VSOR/VRAC via P2YRs in a cell itself and also in a nearby cell in autocrine and paracrine fashions (Fig. 1: *left side (1)* and *(2)*), respectively. From now on, we thus need to answer the question as to what extent swelling-induced ATP release is caused by cell swelling per se and by ATP and glutamate secondary released in the particular cell types under the given conditions. VSOR/VRAC must be activated by BK and S1P released from inflamed cells caused by exposure to extracellular ATP (Fig. 1: *left side, black arrow*), which is one of the danger-associated molecular patterns (DAMPs) causing inflammation in a variety of tissues [81–85], thereby bringing about further VSOR/VRAC activation in the cells stimulated by BK and S1P (Fig. 1: *right* and *left sides (3)*) and therefrom ATP release in a positive feedback manner.

#### VSOR/VRAC-mediated transport of other important negatively charged organic substances

VSOR/VRAC has been shown to serve as conductive pathways also for other negatively charged organic substances, such as GSH, methylene succinic acid or itaconic acid (itaconate), and cGAMP, that are known to be important signaling molecules involved in anti-oxidation, anti-inflammation, and anti-viral defense, respectively.

The most abundant antioxidant GSH is involved in essential cell processes including antioxidant defense, drug detoxification, cell metabolism, and proliferation [86–88]. The release of GSH is a prerequisite to apoptosis induction [89–91]. The first evidence for VSOR/VRAC-mediated GSH release was reported in 2013 by Sabirov et al. [92]. The molecular size of GSH (Table 1) is smaller than the functional pore diameter of VSOR/VRAC (Table 2A). Hypotonicity-induced GSH release from rat thymocytes was largely abolished by a variety of VSOR/VRAC blockers including DCPIB. The VSOR/VRAC permeability to GSH is significant with P_GSH_/P_Cl_ of 0.10 for release from and 0.32 for entry to thymocytes. Subsequently, Friard et al. [93] showed that swelling-induced GSH release is sensitive not only to DCPIB but also to LRRC8A gene knockout in HEK293 cells and that the P_GSH_/P_Cl_ values can be evaluated as 0.08 in HEK293 cells and 0.11 in human kidney tubular epithelial HK2 cells. Even under isotonic conditions, VSOR/VRAC was found to be activated by exposure to TGFβ1, which is a pleiotropic growth factor inducing the epithelial-to-mesenchymal transition (EMT), thereby releasing GSH in HK2 cells [93].

Itaconate, a Krebs cycle-derived metabolite, is produced upon stimulation of Toll-like receptor (TLR) in myeloid cells and is accumulated upon prolonged inflammatory situations. The intracellular itaconate accumulation was shown to inhibit NLRP3 inflammasome activation [94–96]. The itaconate-induced inhibition of NLRP3 inflammasomes was observed to be greatly abolished by myeloid LRRC8A gene knockout [97], suggesting that VSOR/VRAC activity is involved in NLRP3 inflammasome activation, presumably by mediating itaconate efflux. The size of this anti-inflammatory signal, itaconate (C_5_H_6_O_4_), should be smaller than those of glutamate (C_5_H_9_NO_4_) and gluconate (C_6_H_12_O_7_). In fact, the unhydrated diameter of itaconate was calculated to be 6.6 Å as a geometric mean of three dimensions by RZ Sabirov (personal communication). Thus, itaconate is expected to be VSOR/VRAC-permeable. Confirming this inference, Wu et al. [97] showed that hypotonicity induces activation of whole-cell inward currents mediated by efflux of negatively charged itaconate filled in the pipette (intracellular) solution in LPS-primed macrophages and estimated the permeability coefficient of itaconate (P_itaconate_/P_Cl_) for VSOR/VRAC of around 0.2.

cGAMP is an immune-transmitting second messenger produced by cyclic-AMP-GMP synthase (cGAS) in response to cytosolic double-stranded DNAs (dsDNAs) and is an agonist for its receptor, stimulator of interferon genes (STING). cGAMP thereby serves as an important messenger for the cGAS-cGAMP-STING pathway which represents an essential innate immune signaling cascade responsible for the sensing of aberrant cytosolic dsDNA and then plays roles of anti-viral defense and anti-cancer immunity by eliciting interferons (IFNs) [98–100]. Since the size of cGAMP (Table 1) is a little smaller than the effective diameter of VSOR/VRAC pore (Table 2A) estimated by non-electrolyte partitioning [23], VSOR/VRAC channels may mediate cGAMP transport under appropriate conditions. Consistently, the cGAMP uptake/import induced by extracellular application of cGAMP was found to be inhibited by DCPIB in HEK293 cells and primary human umbilical vein endothelial (HUVEC) cells [101], in mouse lung fibroblast (MLF) cells [102, 103], and in murine bone marrow-derived macrophage (BMDM) cells [103]. Also, extracellular cGAMP treatment was found to activate the STING pathway due to VSOR/VRAC-mediated cGAMP import in human lymphoma U937, epithelial HEK293, and endothelial TIME cells incubated in a serum-free isotonic electrolyte solution containing glucose [101]. Gene knockout of LRRC8A suppressed the cGAMP import in HEK293, HUVEC, MLF, and BMDM cells [101–103] as well as in mouse CD4^+^ T cells [104]. Osmotic swelling-induced cGAMP export was electrophysiologically evidenced by the recording of inward currents conveyed by cGAMP^2–^, in a manner sensitive to LRRC8A gene knockdown, in human epithelial HeLa and HCT116 cells [101, 102]. Taken together, it is concluded that VSOR/VRAC is a cGAMP-transporting channel that can mediate bilateral transport of cGAMP. Supporting this conclusion, Zou et al. [102] demonstrated that cGAMP released via VSOR/VRAC channels from host cells infected with DNA viruses is transmitted to distant filter-separated bystander cells and then taken up via VSOR/VRAC channels, in a manner sensitive to LRRC8A gene knockout, by using a trans-well chamber assay in mouse embryonic fibroblasts (MEFs). Thus, it is evident that VSOR/VRAC mediates bilateral transport of cGAMP, especially in association with anti-viral defense immunity.

STING activation induced by extracellular cGAMP application was found to be suppressed by gene knockout not only of LRRC8A but also LRRC8C in U937 and TIME cells [101]. Similarly, LRRC8C gene knockout was observed to inhibit STING activation induced by extracellular cGAMP in CD4^+^ T cells [104]. Thus, VSOR/VRAC responsible for the cGAMP import is likely formed mainly with LRRC8A *plus* LRRC8C. In contrast, STING activation induced by extracellular cGAMP was suppressed by gene knockout of LRRC8A or LRRC8E in BMDM and MLF cells [102, 103]. Also, increased expression of interferon in response to infection with a DNA virus, HSV-1, was inhibited by gene knockout of LRRC8A or LRRC8E but not by triple knockout of LRRC8B, 8C, and 8D genes in MLF cells [102]. Thus, VSOR/VRAC channels formed mainly with LRRC8A *plus* LRRC8E and with LRRC8A *plus* LRRC8C play essential roles in anti-viral immunity [103] and in suppression of the cytotoxic T cell function [104, 105] by bilaterally transporting cGAMP presumably via the channels, respectively. However, further studies are required to precisely determine whether LRRC8 heteromer compositions of cGAMP-transporting VSOR/VRAC vary depending on cell types or cell functions.

## Activation mechanisms of VSOR/VRAC

Activation of VSOR/VRAC was first found to be induced by cell swelling or volume expansion by Hazama and Okada [4] and Cahalan and Lewis [5] in 1988. Thereafter, even without visible cell swelling, VSOR/VRAC was shown to be activated by GTPγS by Doroshenko et al. [106] in 1991 and by a reduction of intracellular ionic strength (Γ_in_) by Cannon et al. [107] and Nilius et al. [108] in 1998. These findings suggest that there are not only a swelling-dependent physiological activation mechanism but also some other swelling-independent activation mechanisms including G-protein-linked biochemical events and Γ_in_-related physicochemical events.

### Swelling-independent physicochemical activation of VSOR/VRAC

**Fig. 2:**
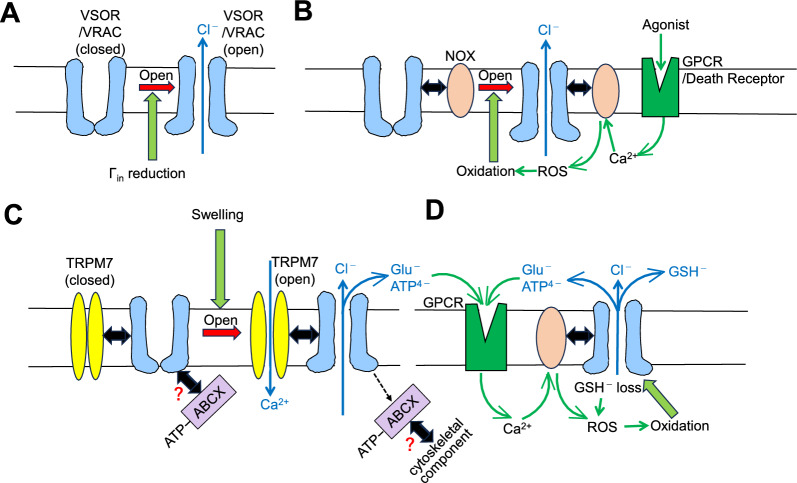
The activation mechanisms of VSOR/VRAC. **A** Swelling-independent activation physicochemically induced by the Γ_in_ reduction. **B** Swelling-independent activation biochemically induced by oxidation due to NOX-mediated ROS production in response to activation of GPCRs and death receptors. **C** First-phase swelling-induced ATP-dependent activation in association with swelling-triggered activation of TRPM7 which physically interacts with LRRC8A. Here, the ATP dependence is assumed to be granted by some ATP-bound ABC protein (here called ABCX) which is assumed to physically interact with VSOR/VRAC molecules, presumably at the convex outside of the LRRD of LRRC8A, but released therefrom upon osmotic swelling followed by an interaction with some cytoskeletal component. **D** Second-phase swelling-induced ROS-dependent activation due not only to NOX-mediated ROS production after GPCR stimulation induced by glutamate (Glu^−^) and ATP^4−^ released as a result of first-phase activation of VSOR/VRAC but also to the elevation of cytosolic ROS level as a result of the loss of intracellular GSH caused by VSOR/VRAC-mediated GSH^−^ release. (See text for details.)

In intact cell systems, VSOR/VRAC activation in the absence of cell swelling was shown to be induced by a large reduction (down to around 30 to 60%) in Γ_in_ [107–110]. After the identification of LRRC8 members as the pore-forming core components [9, 10], similar low Γ_in_-induced channel activation was found in the cell-free reconstitution system formed with purified LRRC8A and LRRC8C, 8D, or 8E in lipid droplet bilayers [111]. However, it is noted that the properties of channels reconstituted in droplet bilayers are different from native VSOR/VRAC, as follows. The reconstituted heteromeric LRRC8 channels are not activated by inflation (volume increase) of droplets but activated by a reduction of Γ_in_ in a manner independent of intracellular ATP and do not exhibit voltage-dependent inactivation kinetics. The homomeric LRRC8A channels reconstituted in liposomes were also found to be activated only in low Γ_in_ solutions [31]. The channel activity was observed even in the absence of ATP and in the presence of a high concentration of free Mg^2+^ on the intracellular side, in contrast to the phenotypical properties of native VSOR/VRAC existing in living cells (see Table 1 in Part 1 article [12]). Since the reduction in Γ_in_ should increase the surface potential on the peripheral surface of highly charged domains of channel-forming proteins, physicochemical/electrostatic repulsion or attraction would take place between any pairs of closely adjacent charged domains or proteins. Deneka et al. [28] suggested that the hydrophilic leucine-rich repeats (LRR) domains (LRRDs: see Fig. 2 in the Part 1 article [12]) are involved in the low Γ_in_-induced activation of VSOR/VRAC, because many basic (negatively charged) and acidic (positively charged) residues exist on the molecular surface of cytoplasmic LRRD. In association with the activation of LRRC8A channels, such physicochemical conformational changes in LRRDs were recently observed [112] by using five synthetic nanobodies called sybodies (sbs): namely, LRRC8A channels expressed in Lrrc8-knockout (LRRC8^−/−^) HEK293 cells were found to be activated by sb4 and sb5 but inhibited by sb1, sb2, and sb3, the former two sbs and latter three sbs of which were shown to bind to the concave inside and convex outside, respectively, of the horseshoe-shaped LRRDs by cryo-EM. On the other hand, the involvement of unfolding of the *N*-terminal (NT) domain of LRRC8 in the VSOR/VRAC activation induced by a large reduction (down to 33%) in Γ_in_ was suggested by Liu et al. [32], mainly based on the molecular dynamics (MD) simulations of cryo-EM structure of LRRC8A. Thus, it can be concluded that VSOR/VRAC is physicochemically activated by the reduction in Γ_in_ through the conformational change in LRRC8 proteins in a manner independent of cell swelling or membrane expansion (Fig. 2A). However, the question as to which domains of LRRC8 proteins are conformationally affected by the Γ_in_ reduction to activate VSOR/VRAC remains to be precisely elucidated.

It must, however, be pointed out that this physicochemical activation mechanism cannot principally account for the swelling-induced activation mechanism because such large extents of Γ_in_ reduction required for VSOR/VRAC activation are unlikely to occur under physiological conditions. Furthermore, cell swelling is known to activate VSOR/VRAC even under constant Γ_in_ conditions [113–116].

### Swelling-independent oxidation-induced activation of VSOR/VRAC

In 2004, hydrogen peroxide (H_2_O_2_), one of the reactive oxygen species (ROS), was found to activate VSOR/VRAC currents under iso-osmotic conditions without leading to cell swelling independently by Shimizu et al. [117] in HeLa cells, Varela et al. [118] in HCT and HeLa cells, and Browe and Baumgarten [119] in cardiomyocytes. This fact was subsequently confirmed by many groups in a variety of cell types [50, 120–133].

Furthermore, Shimizu et al. [117], for the first time, demonstrated that both a mitochondrion-mediated apoptosis inducer staurosporine (STS) and a death receptor-mediated apoptosis inducer tumor necrosis factor-α (TNFα) rapidly activate VSOR/VRAC currents under isotonic conditions in association with significant production of ROS in HeLa cells. Similarly, an ER stress-mediated apoptosis inducer tunicamycin was later shown to increase ROS production and thereby activate VSOR/VRAC currents in rabbit chondrocytes under isotonic conditions [129]. On the other hand, Browe and Baumgarten [119] showed that isotonic VSOR/VRAC activation is induced by angiotensin II via G protein-coupled receptor (GPCR) angiotensin receptor type 1 (AT_1_), the activation of which is known to induce ROS generation in rabbit ventricular myocytes [134]. GPCR-mediated isotonic VSOR/VRAC activation was also shown to be associated with increased ROS production through B2R activation in mouse astrocytes [50, 51], endothelin-1 ET_A_ receptor activation in rabbit atrial and ventricular myocytes [125], and S1PR1 activation in murine RAW 264.7 macrophages [58]. However, the exact molecular mechanism of GPCR-mediated VSOR/VRAC activation is still elusive.

A variety of other chemical stimuli have been shown to elicit ROS production thereby activating VSOR/VRAC currents under isotonic conditions, including a glucan zymosan in rat microglia [123], HIV protease inhibitors in rabbit ventricular myocytes and LL1 cardiomyocytes [125], a brief acid exposure in mouse nodose ganglia neurons [133], and sub-micromolar ouabain in cancer HT-29, KB, and HepG2 cells [135]. Zinc pyrithione (ZPT), which is known to stimulate ROS production [136, 137], was also found to induce VSOR/VRAC activation in the absence of cell swelling in HEK293 cells [138].

It appears that NADPH oxidase (NOX), which generates superoxide and other downstream ROS, is involved in swelling-independent VSOR/VRAC activation, under normal Γ_in_ conditions, in light of the following observations. First, NOX1 was demonstrated to physically interact not only with LRRC8A [139] but also with LRRC8C and 8D [140]. Second, a NOX inhibitor DPI abolished VSOR/VRAC currents induced by STS [117] and β-integrin stretch [119]. Third, a cell-permeable NOX blocker gp9/de-tat was shown to inhibit EGF-induced VSOR/VRAC activation in cardiac myocytes [141]. Fourth, an inhibitor of NOX assembling, 4-(2-aminoethyl)-benzene sulfonyl fluoride (AEBSF), markedly suppressed VSOR/VRAC currents triggered by β-integrin stretch [119]. Since it is known that activation of NOX requires the phosphorylation of its subunit p47phox [142] by PKC [143], VSOR/VRAC activation may also be induced by other chemical agonists for Gq-coupled receptors, for example, a P2YR agonist ATP [48, 144] and a mGluR agonist glutamate [49] in astrocytes through NOX-mediated ROS production presumably triggered by a local intracellular Ca^2+^ rise in the vicinity of Ca^2+^-permeable cation channels, called Ca^2+^ nanodomain [48, 49, 51]. Taken together, it is evident that VSOR/VRAC is biochemically activated by oxidation through NOX-mediated ROS production caused by activation of GPCRs and death receptors, as schematically depicted in Fig. 2B.

Then, the next question is how ROS activate VSOR/VRAC. One possibility is that ROS directly oxidize LRRC8 members thereby inducing some conformational changes in LRRC8. Using *Xenopus* oocytes overexpressed with fluorescently tagged LRRC8 proteins, Pusch and his collaborators found that LRRC8A/8E heteromeric channels were dramatically activated by oxidation [145] through the disulfide bond formation between two cysteines, C424 of LRR1 and C448 of LRR2, in the intracellular LRR regions of LRRC8E [146]. In contrast, they found that LRRC8A/8C and LRRC8A/8D heteromeric channels were rather inhibited by oxidant chloramine-T [145] and by the oxidation of the start methionine (M1) in LRRC8C [146]. In agreement with these observations, VSOR/VRAC currents were inhibited by oxidation in Jurkat T lymphocytes which exhibit a low expression of LRRC8E [145]. However, ROS were, in contrast, found to activate VSOR/VRAC currents in HeLa cells [117, 118] and KB cells [135], both of which express LRRC8D mRNA at much higher levels (around 13.5 and 11.1 times, respectively) than that of LRRC8E mRNA [147], Thus, it seems likely that sensitivity of LRRC8 members to ROS is different from each other depending on cell types and/or experimental conditions. Another possibility is that ROS indirectly lead to the opening of the VSOR/VRAC pore via some second messengers because ROS are known to stimulate a variety of intracellular mediators including several protein kinases and G-proteins [148], some of which have been suggested to regulate VSOR/VRAC activity (see Reviews [7, 8]). Intracellular ATP is expected to be essential for GPCR- and protein kinase-mediated VSOR/VRAC activation. However, it has yet to be determined how ROS activate VSOR/VRAC and whether intracellular ATP is required for ROS-induced VSOR/VRAC activation.

As described in the preceding section, TGFβ1 can activate VSOR/VRAC under isotonic conditions [93]. Also, swelling-independent activation of VSOR/VRAC was recently found to be induced by stimulation not only with TNFα but also with another cytokine IL-1β and some other heat-labile serum protein, in an additive fashion, under isotonic conditions in a manner sensitive to gene knockout of LRRC8A or LRRC8E [103]. This activation was shown to be dependent on the plasmalemmal expression of cGAS which exhibits a physical interaction with LRRC8A [103]. However, it is not known how TGFβ1 and IL-1β induce VSOR/VRAC activation as well as which heat-labile serum protein, other than TNFα and IL-1β, can activate VSOR/VRAC.

### Swelling-induced activation of VSOR/VRAC

Osmotic swelling-induced VSOR/VRAC currents were reported to be only partially inhibited by NOX inhibitors; that is, around 35% suppression by AEBSF in human neutrophils [149] and around 40% inhibition by DPI in mouse astrocytes [49]. Furthermore, swelling- and ROS-induced activation mechanisms were elucidated to be independent of each other, since hypotonicity- and chloramine-T-induced VSOR/VRAC currents observed in *Xenopus* oocytes overexpressed with LRRC8A and 8E were additive [145]. Thus, it appears that a major component of swelling-induced VSOR/VRAC currents is independent of ROS.

#### First-phase ROS-independent, cytosolic ATP-dependent component of swelling-induced activation

As summarized in the Part 1 article [12], TRPM7, which was shown to exert as a mechano-sensitive swelling-activated cation channel by Numata et al. [150, 151], serves as the swelling-sensing subcomponent of VSOR/VRAC not only by enhancing LRRC8A mRNA expression via steady-state Ca^2+^ influx but also by exhibiting real-time functional coupling to VSOR/VRAC activity with showing a physical interaction to LRRC8A protein [11]. It is likely that the TRPM7-mediated Ca^2+^ influx is somehow implicated in the VSOR/VRAC activation caused by cell swelling or membrane expansion. Although a global rise of the intracellular free Ca^2+^ concentration ([Ca^2+^]_i_) is not required for swelling-induced VSOR/VRAC activation (see Review [7]), there remains a possibility of an involvement of localized Ca^2+^ rise therein [49, 152]. In any case, it is evident that hypotonicity-induced VSOR/VRAC is associated with conformational changes in pore-forming LRRC8 proteins, because this activation was observed to be coupled to the displacement of *C*-terminal LRRDs in HeLa cells and LRRC8^−/−^ HEK293 cells transfected with fluorescence-labeled LRRC8A and LRRC8E by FRET studies [153].

Although cytosolic ATP dependence is one of the most important physiological properties of VSOR/VRAC, its exact molecular mechanism is still missing, as pointed out in the previous review articles [7, 12]. Non-hydrolytic requirement of intracellular ATP may suggest that some ATP-binding protein plays an essential role in the mechanism of VSOR/VRAC activation. In agreement with this inference, so far, four members of the ATP-binding cassette (ABC) transporter superfamily proteins, three of which are membrane-spanning and another is cytosolic proteins, have been reported to be involved in the regulation of VSOR/VRAC activity. The chronologically first one is P-glycoprotein (PGP), the MDR1 gene product, which was initially proposed as the molecular entity of VSOR/VRAC [154, 155]. Although its “PGP = VSOR/VRAC” hypothesis was later rejected [156], PGP was shown to upregulate the volume sensitivity of VSOR/VRAC channel [157]. Second, CFTR which has a structural similarity to PGP and exerts as the cAMP/PKA-dependent Cl^−^ channel, was shown to downregulate VSOR/VRAC currents [158, 159] through the second nucleotide-binding domain (NBD2) [159]. The third one ABCF2, a cytosolic member of the ABC proteins, was shown to suppress VSOR/VRAC activity [160], as detailed below. The last one ABCG1, a cholesterol-exporting ATPase, was shown to enhance hypotonicity-induced ATP release mediated by VSOR/VRAC presumably through reduction of the cholesterol level within the plasma membrane [73]. Depletion of membrane cholesterol content was shown to enhance VSOR/VRAC activation induced by mild hypotonic stimulation [161–163]. Subsequently, cholesterol depletion-induced VSOR/VRAC activation was demonstrated to be mediated by F-actin [164], the expression of which was recently shown to be essential for VSOR/VRAC activity [165]. Since cholesterol depletion was reported to release several cytoskeletal proteins, such as actin, α-actinin, and ezrin from the cellular membrane fractions [166], there arises a possibility that an interaction between an ATP-binding protein and a cytoskeletal component released in response to osmotic cell swelling is involved in the swelling-induced activation mechanism of VSOR/VRAC. Taken together, swelling-induced activation of VSOR/VRAC is likely regulated by TRPM7 and some ABC proteins, which may interact with LRRC8 members as well as some cytoskeletal components in the native cell system, as schematically drawn in Fig. 2C.

Ando-Akatsuka et al. [160] found that an actin-binding and -crosslinking protein, α-actinin-4 (ACTN4), which is ubiquitously expressed in non-muscle cells [167] and participates in the cytoskeleton organization [168, 169], becomes associated with the plasma-membrane upon osmotic cell swelling. Next, by the protein overlay assays combined with proteomics approaches, a cytosolic member of the ABC transporter protein superfamily, ABCF2, was identified as the binding partner of ACTN4, and then the physical interaction (binding in the broad sense) between ACTN4 and ABCF2 was found to be prominently enhanced by hypotonic cell swelling. Furthermore, knockdown and overexpression of ABCF2 were shown to augment and suppress the VSOR/VRAC activity, respectively. Therefore, it was concluded that swelling-induced activation of VSOR/VRAC is accomplished by the protein–protein interaction between ACTN4 and ABCF2, thereby preventing ABCF2 from inhibiting VSOR/VRAC activity. Thus, it is likely that the cytosolic ATP-binding protein ABCF2 represents an endogenous blocking subcomponent of VSOR/VRAC. ABCF2 may also grant non-hydrolytic ATP dependence and free Mg^2+^ sensitivity to VSOR/VRAC, if only the form of ABCF2 bound to ATP, but not to Mg-ATP, can be released via VSOR/VRAC, thereby activating VSOR/VRAC upon osmotic swelling. Further investigations are warranted to prove this inference by testing the possibility that ABCF2 physically interacts with or directly binds to LRRC8 member proteins, especially to LRRDs which were shown to be required for swelling-induced VSOR/VRAC activation [170].

#### Second-phase ROS-dependent, GPCR-mediated component of swelling-induced activation

Hypotonic stimulation has been often found to bring about ROS production under certain conditions [118, 128, 129, 133, 171, 172]. However, this fact does not necessarily imply a direct action of osmotic swelling. As described in the preceding section, osmotic cell swelling often induces VSOR/VRAC-mediated release of GSH, glutamate, and ATP depending on cell type. Therefore, osmotic swelling may indirectly result in a rise of intracellular ROS level caused by GSH release mediated by VSOR/VRAC and by ROS production due to GPCR activation induced by glutamate and ATP. Consistently, hypotonicity-induced ROS production was shown to be mediated by NMDA receptors in rat astrocytes [173]. Thus, it is conceivable that swelling-induced VSOR/VRAC activity is enhanced by GPCR-mediated ROS production and VSOR/VRAC-mediated GSH^−^ release in a manner of positive feedback control, as schematically depicted in Fig. 2D. However, it must be noted that this component is the secondary result of earlier ROS-independent swelling-induced VSOR/VRAC activation (Fig. 2C).

Swelling-induced VSOR/VRAC activity was observed to be upregulated by an increase in intracellular cAMP through adenylate cyclase (AC)-coupled Ca^2+^-sensing receptor, CaR, and arginine vasopressin type-2 receptor, V2R, both of which belong to the Gs-coupled receptor family, in response to elevation of extracellular Ca^2+^ [174] and arginine vasopressin [175], respectively. Stimulation of protein-tyrosine kinase (PTK)-coupled epidermal growth factor receptor, EGFR, was also shown to upregulate swelling-induced VSOR/VRAC activity [176]. The exact upregulating mechanisms of cAMP/AC- and PTK-mediated signaling pathways remain elusive.

## Conclusions and perspectives

The volume-sensitive outwardly rectifying/volume-regulatory anion channel (VSOR/VRAC) activated by cell swelling transports inorganic halide anions (mainly Cl^−^), thereby regulating the cell volume after osmotic swelling. In addition, this channel was shown to serve as transporting pathways for many organic substances, the sizes of which are smaller than the VSOR/VRAC pore size. These organic substances include major extracellular messenger molecules for autocrine/paracrine signaling such as glutamate and ATP as well as anti-oxidant GSH, anti-inflammatory itaconate, and anti-viral defensing cGAMP. The activation mechanisms of VSOR/VRAC are classified into swelling-dependent and -independent ones. Reduction of intracellular ionic strength (Γ_in_) physicochemically activates VSOR/VRAC due to the conformational changes in LRRC8 proteins in a manner independent of cell swelling. Also, VSOR/VRAC can be biochemically activated by oxidation even in the absence of cell swelling, because LRRC8 proteins are physically interacting with NOX which releases ROS, when some GPCRs and death receptors are activated. The mechanisms of swelling-induced activation are composed of two phases. The first phase is dependent on swelling-sensing TRPM7 which exhibits a physical interaction with the LRRC8A molecule and on the nonhydrolytic existence of intracellular free ATP. The second phase is dependent on GPCR activation triggered by glutamate and ATP which are released via VSOR/VRAC activated in the first phase and on the ROS production due to GPCR-mediated NOX activation and GSH release via VSOR/VRAC activated in the first phase. After the identification of the pore-forming core components of VSOR/VRAC as LRRC8 members, a large number of recent studies have elucidated the molecular processes of VSOR/VRAC-mediated release of organic substances and of VSOR/VRAC activation. However, still much remains unanswered, and many new questions have arisen, as pointed out in each section of this article and collectively listed in Supplementary Table as research subjects that remain to be studied in the near future.

Since VSOR/VRAC was recently demonstrated to be activated by inflammatory signals, BK and S1P, as well as by anti-inflammatory signals, itaconate and cGAMP, there arises a possibility that VSOR/VRAC activity plays some important and reciprocal roles in the inflammation processes. Further studies are warranted to investigate this possibility.

### Supplementary Information

Additional file 1.

## Data Availability

The data underlying this article will be obtained via PubMed and Google Scholar or available from the author upon reasonable request.

## Abbreviations

CFTR: Cystic fibrosis transmembrane conductance regulator
VAAC: Volume-activated anion channel
VSOR: Volume-sensitive outwardly rectifying anion channel
VRAC: Volume-regulated anion channel
Maxi-Cl: Large-conductance ohmic maxi-anion channel
VSOR/VRAC: The volume-sensitive outwardly rectifying/volume-regulatory anion channel
VSOAC: Volume-sensitive organic osmolyte and anion channel
cryo-EM: Cryo-electron microscopy
GSH: Glutathione
cGAMP: 2′3′-Cyclic-GMP-AMP
mGluR: Muscarinic glutamate receptor
P2YR: Purinergic P2Y receptor
BK: Bradykinin
B2R: Bradykinin B2 receptor
S1P: Sphingosine-1-phosphate
DAMP: Danger-associated molecular pattern
EMT: Epithelial-to-mesenchymal transition
TLR: Toll-like receptor
cGAS: Cyclic-AMP-GMP synthase
dsDNA: Double-stranded DNA
STING: Stimulator of interferon genes
IFN: Interferon
MLF: Mouse lung fibroblast
BMDM: Bone marrow-derived macrophage
MEF: Mouse embryonic fibroblast
Γ_in_: Intracellular ionic strength
LRR: Leucine-rich repeat
NT: N-terminal
MD: Molecular dynamics
sbs: Sybodies
ROS: Reactive oxygen species
STS: Staurosporine
TNFα: Tumor necrosis factor-α
GPCR: G protein-coupled receptor
AT_1_: Angiotensin receptor type 1
S1PR1: Sphingosine-1-phosphate receptor 1
ZPT: Zinc pyrithione
NOX: NADPH oxidase
AEBSF: 4-(2-Aminoethyl)-benzene sulfonyl fluoride
ABC: ATP-binding cassette
PGP: P-glycoprotein
NBD2: Second nucleotide-binding domain
AC: Adenylate cyclase
CaR: Ca^2+^-sensing receptor
PTK: Protein-tyrosine kinase
