# Supplementary material for: Physiology of the volume-sensitive/regulatory anion channel VSOR/VRAC: part 2: its activation mechanisms and essential roles in organic signal release
Source: J Physiol Sci. 2024 Jun 14;74:34. doi: 10.1186/s12576-024-00926-3 (PMC11177392; doi:10.1186/s12576-024-00926-3)
Supplement: Supplementary file 1 — Additional file 1. [file 12576_2024_926_MOESM1_ESM.docx]

**Supplementary Table. Research subjects that remain to be investigated in the near future**

A) Regarding VSOR/VRAC-mediated release of organic substances

1. Evaluation of the pore sizes of LRRC8A/8D and LRRC8A/8E heteromers under cryo-EM observations.

2. Differential estimation of the contributions of VSOR/VRAC and Maxi-Cl to swelling-induced release of glutamate/ATP from the given cell types including astrocytes.

3. Differential evaluation on the contribution of cell swelling *per se* and the contributions of glutamate and ATP secondary released via VSOR/VRAC to swelling-induced release of glutamate/ATP in the given cell types including astrocytes.

4. Differential evaluation of the suppressing effect caused by the open-channel blocking action of released ATP to VSOR/VRAC and the enhancing effect caused by the receptor-mediated augmenting action of released ATP to VSOR/VRAC on swelling-induced glutamate/ATP release via VSOR/VRAC.

5. Determination of variation in the LRRC8 heteromer compositions of cGAMP-transporting VSOR/VRAC depending on cell types and on cell functions.

B) Regarding the activation mechanisms of VSOR/VRAC

1. Elucidation of the exact domains of LRRC8 proteins that are conformationally affected by the Γ_in_ reduction to activate VSOR/VRAC.

2. Clarification about the exact molecular mechanism of GPCR-mediated VSOR/VRAC activation by glutamate and ATP.

3. Clarification about the mechanism of ROS-induced VSOR/VRAC activation and therein role of intracellular ATP.

4. Clarification about the mechanisms of VSOR/VRAC activation by TGFβ1and IL-1β.

5. Identification of the heat-labile serum protein, other than TNFα and IL-1β, that can activate VSOR/VRAC.

6. Clarification about the exact molecular mechanism for nonhydrolytic dependence of VSOR/VRAC on cytosolic ATP.

7. Verification of the hypothesis that the ATP-bound form of ABCF2 directly interacts with LRRC8 member protein, thereby maintaining VSOR/VRAC in the closed state.

8. Clarification about the exact mechanisms for VSOR/VRAC upregulation evoked by AMP/AC- and PTK-mediated signaling pathways.
